# Haemotoxic snake venoms: their functional activity, impact on snakebite victims and pharmaceutical promise

**DOI:** 10.1111/bjh.14591

**Published:** 2017-02-24

**Authors:** Julien Slagboom, Jeroen Kool, Robert A. Harrison, Nicholas R. Casewell

**Affiliations:** ^1^ Alistair Reid Venom Research UnitParasitology DepartmentLiverpool School of Tropical MedicineLiverpoolUK; ^2^ Division of BioAnalytical ChemistryDepartment of Chemistry and Pharmaceutical SciencesFaculty of SciencesVrije Universiteit AmsterdamAmsterdamThe Netherlands

**Keywords:** envenoming, neglected tropical diseases, toxin, haemorrhage, venom‐induced consumption coagulopathy

## Abstract

Snake venoms are mixtures of numerous proteinacious components that exert diverse functional activities on a variety of physiological targets. Because the toxic constituents found in venom vary from species to species, snakebite victims can present with a variety of life‐threatening pathologies related to the neurotoxic, cytotoxic and haemotoxic effects of venom. Of the 1·8 million people envenomed by snakes every year, up to 125 000 die, while hundreds of thousands survive only to suffer with life‐changing long‐term morbidity. Consequently, snakebite is one of the world's most severe neglected tropical diseases. Many snake venoms exhibit strong haemotoxic properties by interfering with blood pressure, clotting factors and platelets, and by directly causing haemorrhage. In this review we provide an overview of the functional activities of haemotoxic venom proteins, the pathologies they cause in snakebite victims and how their exquisite selectivity and potency make them amenable for use as therapeutic and diagnostic tools relevant for human medicine.

Venomous snakes are a species‐rich (~2500) group of squamate reptiles that are widely distributed across most of the Earth's landmasses and many of its oceans (Greene, 1997). These snakes primarily use venom for facilitating prey capture, although when threatened or provoked they use venom defensively, as observed in cases of human snakebite. The snake venom system consists of a pair of venom secretory glands, found either side of the head on the upper jaw, that are connected to ducts which transmit venom from the gland to the base of modified teeth (fangs) used for injection (Kerkkamp *et al*, 2015). Despite much morphological variation observed in the venom system of these animals, particularly pertaining to the structure of venom glands and the morphology and dental location of fangs, developmental and molecular evidence suggests that these structures are homologous and that venom originated on one occasion in snake ancestors at least 60 million years ago (Fry *et al*, 2008; Vonk *et al*, 2008; Vidal *et al*, 2009; Kerkkamp *et al*, 2015).

Despite this common evolutionary origin, we find considerable variation in the toxic constituents found in the venom of different snake species (Chippaux *et al*, 1991; Fry *et al*, 2008). Venoms consist of complex mixtures of proteinacious components (circa 50–200), which are commonly referred to as toxins. These toxins have evolved from a number of non‐toxic housekeeping genes by a variety of processes, most commonly gene duplication followed by neofunctionalization under the action of positive selection, but also via alternative splicing, recombination and simple modification of expression (Cousin *et al*, 1998; Lynch, 2007; Casewell *et al*, 2011; Moura‐da‐Silva *et al*, 2011; Vonk *et al*, 2013). Over millions of years of evolutionary time these ancestral venom toxins have diversified further via subsequent gene duplication events, ultimately giving rise to related toxin isoforms that are encoded by the same multi‐locus gene family, but which often have distinct and/or complimentary functional activities (Fry *et al*, 2003; Lynch, 2007; Casewell *et al*, 2011; Vonk *et al*, 2013). Crucially, the rapid rate at which snake venom toxins have evolved, and their lineage‐specific gene duplication and loss events, result in different snake species having different venom toxins. Consequently, variation in venom composition is observed across all taxonomic levels of snakes, between families, genera, species and even over the lifetime of an individual (Chippaux *et al*, 1991; Durban *et al*, 2013; Casewell *et al*, 2014). A schematic overview of how variation in snake venom composition has evolved is presented in Fig 1.

**Figure 1:**
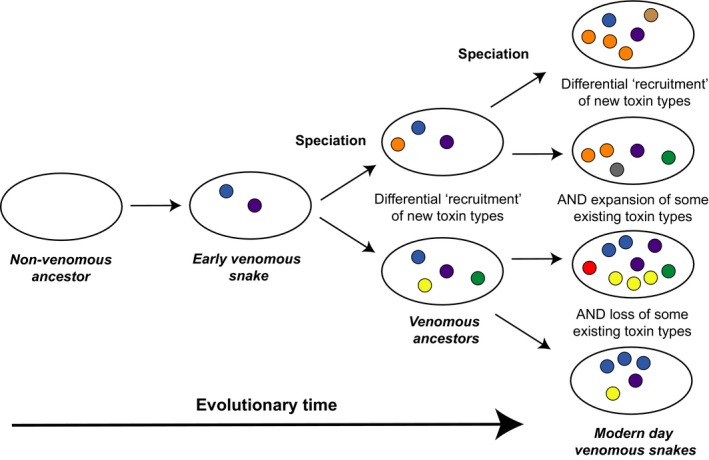
A simplified schematic demonstrating how snake venoms have evolved into variable toxin mixtures over evolutionary timescales. Ovals represent venom glands in different snakes (hypothetical ancestors or modern day species) and coloured circles represent different toxin types. Non‐venomous ancestors had no toxins in their venom gland (or expressed them at very low levels), but early venomous snake ancestors recruited a number of toxin types into their venom. Over evolutionary time, as lineages diverged, different toxin types became utilised in the venom of certain snakes, and these toxin types diversified differentially via the process of gene duplication and loss, resulting in variation in venom composition observed between different groups of snakes.

Ultimately, these variable toxin components underpin the functional bioactivity of venom. Snake venoms can be broadly classified as haemotoxic, neurotoxic or cytotoxic (World Health Organization, 2010a). Many species possess multi‐functional venoms that contain toxins capable of inducing different toxicities and, while there are general rules as to what to expect from human envenomings by many species, there are also many exceptions. For example, the two major medically important snake families are the vipers (family Viperidae; e.g. rattlesnakes, adders, vipers) and the elapids (family Elapidae; e.g. cobras, mambas, coral snakes) (Fig 2). A simplified historical overview is that viper venoms are predominately haemorrhagic and elapid venoms neurotoxic. While this simplification holds for many species there are a number of examples of vipers causing neurotoxicity (Silva *et al*, 2017) and elapids causing bleeding disturbances (Berling *et al*, 2015). Consequently, venom variation is a crucial factor that significantly impacts upon snakebite pathology and the treatment of snakebite.

**Figure 2:**
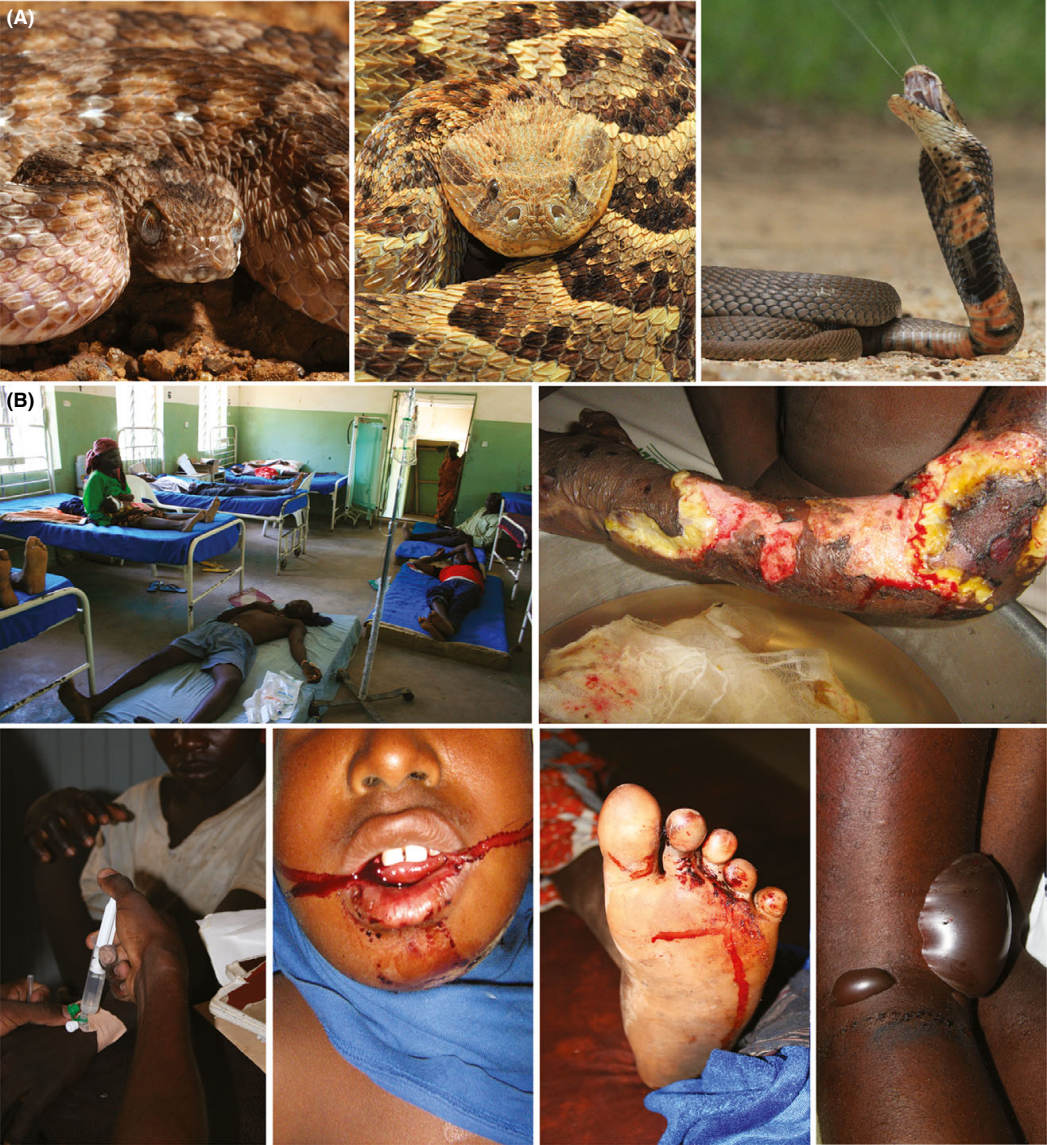
Representative venomous snakes and the pathology they cause in sub‐Saharan Africa. (A) Examples of medically‐important venomous snakes. From left: Joger's saw‐scaled viper (*Echis jogeri*) from Senegal; the puff adder (*Bitis arietans*) from Kenya; the Mozambique spitting cobra (*Naja mossambica*) from South Africa. Photographs courtesy of Wolfgang Wüster. (B) Pathology caused by venomous snakes. Clockwise from top left: A dedicated ward for snakebite victims in Kaltungo, Nigeria; extensive local tissue damage following a bite by an unknown cytotoxic snake (probably the spitting cobra *Naja nigricollis*) in north‐east Nigeria; blood filled blisters following a bite by the cytotoxic puff adder (*Bitis arietans*) in the area of Kilifi, Kenya; local and systemic haemorrhage (two images) facilitated by venom‐induced consumption coagulopathy following bites by saw‐scaled vipers (*Echis ocellatus*) in north‐east Nigeria; a patient receiving a slow intravenous infusion of antivenom following a bite by a saw‐scaled viper (*E. ocellatus*) in north‐east Nigeria. Photographs courtesy of the Alistair Reid Venom Research Unit, Liverpool School of Tropical Medicine.

## Snakebite is a neglected tropical disease

Venomous snakes bite up to 5·5 million people every year (Kasturiratne *et al*, 2008). These bites are responsible for causing as many as 1·8 million envenomings and 125 000 deaths annually, with three to five times that number of people thought to suffer from long‐term morbidity (Chippaux, 1998; Kasturiratne *et al*, 2008; Habib *et al*, 2015). Consequently, snakebite is one of the world's most lethal neglected tropical diseases. Despite this, snakebite receives little attention from global health agencies, charities or governments, perhaps best exemplified by the World Health Organization (WHO) currently omitting snakebite from their formal list of neglected tropical diseases (http://www.who.int/neglected_diseases/diseases/en/). Despite this, the snakebite community continues to lobby for greater recognition surrounding this issue (Williams, 2015; Arnold, 2016), and recent progress has led to the WHO formally considering recognising snakebite as a neglected tropical disease at the upcoming World Health Assembly in 2017 (Harrison & Gutiérrez, 2016).

**Figure 3:**
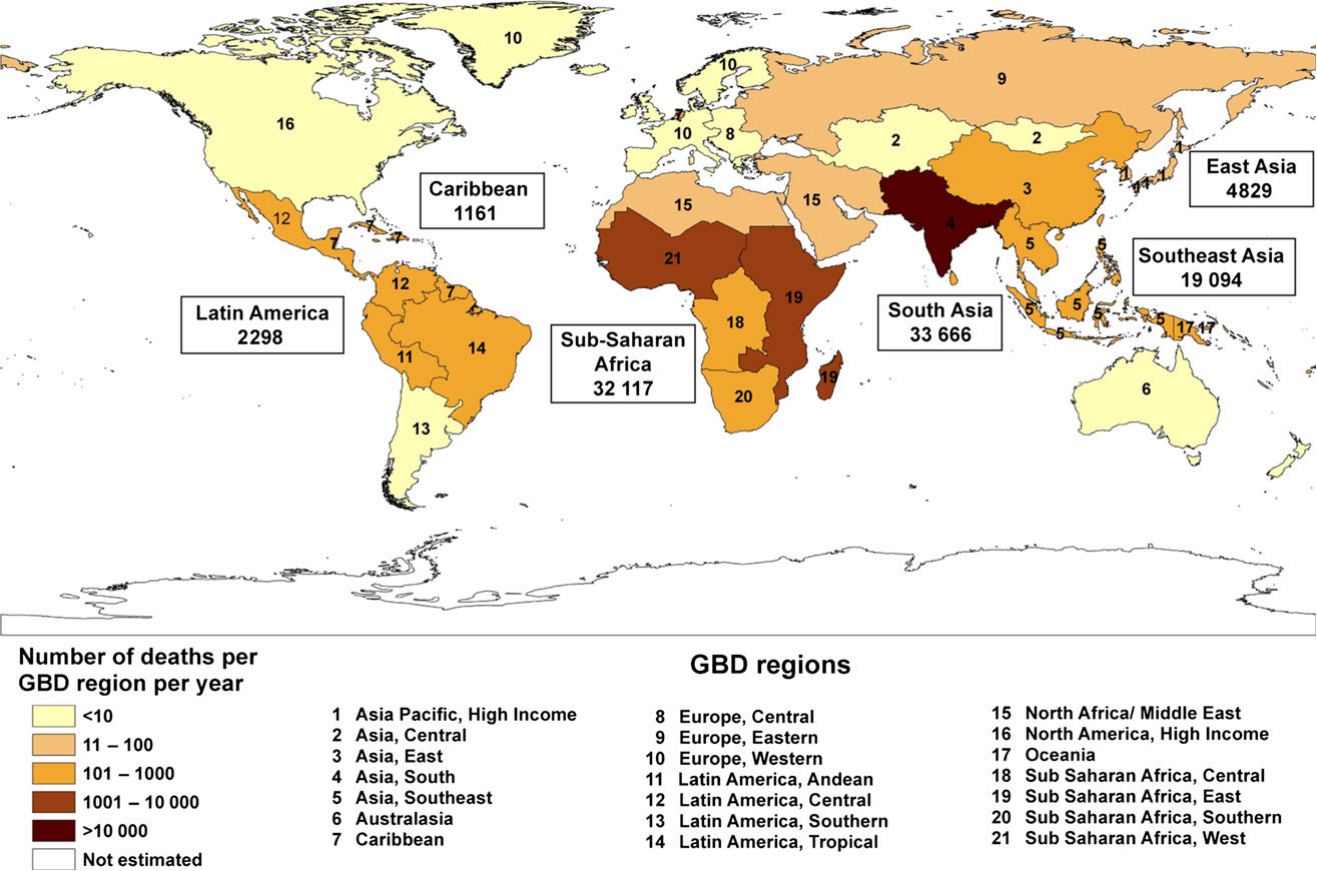
Estimates of annual deaths caused by snakebite in different global burden of disease (GBD) regions. Conservative estimates are displayed by different colour shadings on the map and upper estimates are annotated for each major region where the number of snakebite deaths is greater than 1000. The world map image is reproduced from Kasturiratne *et al,* (2008)under the Creative Commons Attribution License and the overlaid upper estimates of snakebite death are taken from the same publication.

Snakebite predominately affects people living in the tropical and sub‐tropical regions of the world. While venomous snakes inhabit every continent, the vast majority of deaths occur in sub‐Saharan Africa and South and South‐East Asia (Fig 3) (Kasturiratne *et al*, 2008). Males are bitten more often than females, and bites are most common in younger adults and children, with children and the elderly seemingly suffering the greatest risk of mortality (World Health Organization, 2010b). Fundamentally, snakebite most greatly affects the rural impoverished people of the tropics (Harrison *et al*, 2009). There is a significant association between snakebite‐induced mortality and (i) the human developmental index, (ii) the percentage of the labour force working in agriculture, (iii) the per capita government expenditure on health and (iv) the gross domestic product (GDP) per capita of a country (Harrison *et al*, 2009). In part these associations are due to the rural people of the tropics frequently encountering snakes due to working in their habitat, typically in agricultural areas, and therefore are bitten more frequently. But also because the health infrastructure and economic status of these countries is less developed, meaning that victims often cannot afford protective footwear [>80% of bites are on the lower limb (Chippaux, 2010)], they have to travel for many hours to receive treatment (if they seek healthcare at all), and treatments are often not available within their available health infrastructure or, if they are, are unaffordable to many.

The only specific therapy available for treating snakebite is antivenom. Antivenom consists of polyclonal antibodies that are generated by immunising animals (horses or sheep) with small amounts of snake venom. The resulting antibodies are purified from serum or plasma and formulated into intact IgG or F(ab’)_2_‐ or Fab‐fragment therapies, which are administered intravenously following snakebite. While antivenoms are effective therapies for neutralising the toxicity of snake venoms if delivered promptly, there are a number of deficiencies with these products. First is the issue of venom variation, described earlier. The antibodies present in any antivenom are specific to those venoms that were used for immunisation. Whilst these antibodies might cross‐react with and neutralise similar venom toxins present in different species than those used for immunisation, there will be a limit to their cross‐efficacy, and this is often restricted to the same genus of snakes (Williams *et al*, 2011; Tan *et al*, 2016a,b). Despite a number of recent reports suggesting that certain antivenoms might have greater paraspecificity than previously anticipated (e.g. Pla *et al*, 2014; Tan *et al*, 2015), there are also a number of reports describing the poor performance or failure of antivenoms at neutralising venom from snakes related to those used for immunisation (Casewell *et al*, 2010; Segura *et al*, 2010; Tan *et al*, 2016b). Limitations with the paraspecificity of antivenoms are best exemplified by reports from sub‐Saharan Africa where geographically inappropriate products were used to treat snakebite in the Central African Republic and Ghana, resulting in increases in case fatality rates from 0·4% and 1·0% to 10·0% and 12·1% respectively (Visser *et al*, 2008; Alirol *et al*, 2015).

To try and circumvent these limitations, many manufacturers generate antivenoms by immunising animals with multiple different snake venoms. These products have the benefit of generating antibodies against a wider pool of antigens (i.e. the variety of toxins found in different snake venoms) and they also circumvent clinical challenges surrounding identifying the snake that has bitten a patient required to inform antivenom choice. However, the consequences of this approach are that the antivenom contains fewer specific antibodies to the single snake species that envenomed the patient, effectively making them more dilute. Therefore, larger therapeutic doses are required to effect cure (Abubakar *et al*, 2010). This in turn brings two challenges, a potential increased risk of adverse reactions as larger doses of foreign protein are delivered to human victims, and an increased treatment cost as more vials are required to effect cure. Considering that the incidence of adverse reactions reported after antivenom therapy can be as high as 55% (Deshpande *et al*, 2013) and the average cost of a vial of antivenom varies from $100–250 in Africa for example, with upwards of 10–20 vials being required to effect cure (World Health Organization, 2010a), these characteristics are far from ideal. There is therefore an urgent need for the development of new, low‐dose, low‐cost and paraspecifically effective antivenoms for treating snakebite victims in the tropical regions of the world (Harrison *et al*, 2011).

## Snakebite causes a multitude of pathologies

Because of the diversity of toxic components found in the venom of any one snake, the pathological consequences of envenoming can be diverse and multifactorial. Moreover, variables such as the location of the bite site and the amount of venom injected [which in turn can be highly variable (Alirol *et al*, 2010)] can contribute to the severity of clinical signs observed in snakebite victims. While pathology can be limited to local effects surrounding the bite site, such as pain, oedema and bruising, many envenomings also result in systemic pathology, which can be severe and lethal if untreated. Clinical patterns of envenoming can be broadly classified into three groups: neurotoxic, cytotoxic and haemotoxic, although myotoxicity can also present in certain cases (World Health Organization, 2010a). It is important to note that certain snake species are capable of causing combinations of these different toxicities, and there are also examples of the same snake species causing different pathologies in human patients across different parts of their range (Antonypillai *et al*, 2011; Massey *et al*, 2012).

Neurotoxic envenoming is characterised by descending neuromuscular paralysis, beginning with the eyes (ptosis), facial muscles and other muscles innervated by the cranial nerves, before progressing to respiratory and generalised flaccid paralysis (Warrell, 1995a,b; Chippaux, 2010; World Health Organization, 2010a,b). The toxins predominately responsible for neurotoxic venom effects are members of the diverse phospholipases A_2_ (PLA_2_) and three‐finger toxin (3FTX) families (Fry *et al*, 2003; Lynch, 2007). These toxins are capable of acting on the pre‐ and/or post‐synaptic junction, where they can have a multitude of actions, from blocking potassium or sodium channels, to acting as nicotinic or muscarinic receptor antagonists (Fry *et al*, 2003; Lynch, 2007; Casewell *et al*, 2013). Ultimately, many snakes contain multiple different neurotoxins in their venom that perturb neurotransmission at the neuromuscular junction, resulting in paralysis.

Snakebite victims suffering from cytotoxic envenoming are characterised by painful and progressive swelling at the bite site, developing into blistering and bruising, that are sometimes coupled with systemic effects, which include hypovolaemic shock (World Health Organization, 2010a,b). Often, extensive local tissue damage develops (Fig 2), characterised by necrosis of the affected limb and requiring surgical debridement or amputation if left untreated. Hydrolytic enzymes, such as snake venom metalloproteinases (SVMPs) and PLA_2_s, and non‐enzymatic cytotoxic 3FTXs have been implicated as the causative agents found in different snake venoms (Escalante *et al*, 2009; Rivel *et al*, 2016). Recently it was shown that the destruction of local tissue may also be promoted by snake venom inducing the formation of neutrophil extracellular traps (NETs), which in turn block blood vessels and contain the venom toxins to bite site, thereby promoting cytotoxic pathology (Katkar *et al*, 2016).

**Figure 4:**
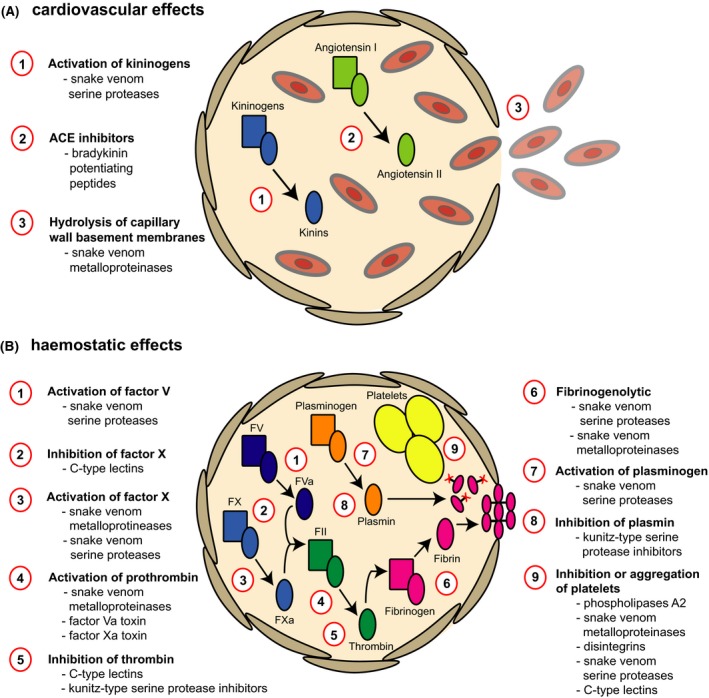
A schematic overview of the physiological targets of haemotoxic snake venom toxins. (A) The targets for venom toxins that cause cardiovascular effects, which present clinically as hypotension. (B) The targets for venom toxins that cause haemostatic effects, which present as coagulopathy. Each physiological target is indicated by a red circle and the venom toxin type(s) that target these sites are listed. ACE, angiotensin‐converting enzyme; FV – Factor V; FVa, activated Factor V; FX, Factor X; FXa, activated Factor X; FII, prothrombin.

For the remainder of this review we focus on haemotoxicity caused by snake venoms. Haemotoxicity is one of the most common clinical signs in victims of snakebite, particularly when viperid snakes are responsible for envenomings. Broadly speaking, haemotoxic venoms can have cardiovascular and/or haemostatic effects. Cardiovascular effects are perhaps best characterised by a dramatic fall in blood pressure and this can be caused by a number of different venom toxins. For example, SVMPs indirectly contribute to hypotension by increasing vascular permeability via the degradation of capillary basement membranes, resulting in leakage and reductions in blood pressure (Gutiérrez *et al*, 2016). Snakes can also directly induce vasodilatory effects via the injection of bradykinin potentiating peptides (BPPs) present in their venom, and this activity can be further enhanced by certain snake venom serine protease (SVSP) toxins exhibiting kallikrein‐like functionalities, causing the release of bradykinins from plasma kininogens (Phillips *et al*, 2010; Camargo *et al*, 2012). Ultimately, these various venom toxins, whether acting alone or in combination, can cause shock in envenomed patients due to systemic hypotension (Fig 4A).

Haemostatic effects caused by snake venoms are best characterised by local and systemic haemorrhage. Overt signs include bleeding from the gums, recently healed wounds, the bite site, the gastro‐intestinal and/or genito‐urinary tracts and/or haematemesis and haemoptysis (Fig 2) (Warrell, 1995a,b; World Health Organization, 2010b). As described above, SVMP toxins increase the vascular permeability of blood vessels by degrading capillary basement membranes, resulting in extravasation (Gutiérrez *et al*, 2016), and therefore these toxins also exert haemorrhagic activities (Fig 4A). While spontaneous systemic bleeding therefore contributes to deaths caused by shock (hypotension), snake venom is also responsible for causing fatalities via haemorrhage, particularly when intracranial bleeding occurs (Mosquera *et al*, 2003).

Haemorrhage caused by snake venom is often complicated and exacerbated by patients presenting with blood clotting disturbances as the result of venom‐induced consumption coagulopathy (VICC). VICC, a disseminated intravascular coagulation (DIC)‐like syndrome, is characterised by low or undetectable levels of fibrinogen, resulting in incoagulable blood (Fig 2) (Isbister, 2010; Maduwage & Isbister, 2014). However, unlike DIC, VICC does not usually result in systemic microthrombi and end‐organ failure and it presents with a rapid onset and resolution (Isbister, 2010). Although the majority of snakes known to cause VICC are vipers, certain elapid snakes from Australasia and colubrid and natricine snakes from Africa and Asia have also been reported to cause consumptive coagulopathies via the action of their procoagulant toxins (Maduwage & Isbister, 2014). These toxins are diverse and include SVMPs, SVSPs and toxic forms of Factor X and Factor V (Phillips *et al*, 2010; Rosing & Tans, 2010; Kini & Koh, 2016). The targets for these toxins are also varied, with many activating clotting factors found towards the end of the clotting cascade, such as Factor X and prothrombin, while others are fibrinogenolytic (Fig 4B) (Maduwage & Isbister, 2014; Kini & Koh, 2016). Consequently, in addition to the depletion of fibrinogen, patients suffering from VICC often exhibit other factor deficiencies due to their consumption, including Factor II, Factor V, Factor VIII and Factor X (Isbister *et al*, 2010, 2015; Maduwage & Isbister, 2014). Although many of the countries where snakebite is abundant rely on simple bedside tests to detect incoagulable blood (e.g. the 20‐min‐ whole blood clotting test), a number of studies have demonstrated that patients suffering from VICC present with a prolonged prothrombin time (PT), and an international normalised ratio and activated partial thromboplastin time (aPTT) that are either very high or exceed the upper limits of detection (Isbister *et al*, 2010, 2015). Finally, many snake venom toxins are known to act on platelets. C‐type lectins, disintegrins and certain metalloproteinases, to name a few, are capable of either inducing the aggregation of platelets, for example by von Willebrand factor‐ or collagen‐mediated activation, or inhibiting their aggregation, by potently blocking integrin receptors found on the surface of platelets, such as α_2_β_3_ (Rucavado *et al*, 2005; Chakrabarty & Chanda, 2015; Kini & Koh, 2016). Both inhibition and activation (via hypoaggregation) result in these toxins contributing to venom‐induced coagulopathies by depleting platelets, and this presents clinically as marked thrombocytopenia (Warrell, 1995a,b; Rucavado *et al*, 2005).

The consequences of venom‐induced coagulopathies are an increased risk of serious and life‐threatening haemorrhage, particularly when envenoming is the result of bites by snakes that also contain haemorrhagic SVMPs in their venom. Indeed, in many fatal cases involving intracranial haemorrhage, snakebite victims were found to also be suffering from clotting disorders (Mosquera *et al*, 2003; Berling *et al*, 2015). Consequently, coagulopathy has been described as the most common, important, systemic, clinical syndrome caused by snake envenoming (Maduwage & Isbister, 2014).

## Haemotoxic snake venom components

In this section we provide an overview of the snake venom toxin types that exhibit haemotoxic properties, with a particular focus on the SVMPs and SVSPs. We summarise the various haemotoxic activities of these toxins in Fig 4. SVMPs are a diverse enzymatic toxin family that are found in the venom of most advanced snakes, although they are most commonly key components in viperid species (Casewell *et al*, 2015). Having diversified extensively via gene duplication followed by the loss of functional domains (Casewell *et al*, 2011), a single snake species can contain more than a dozen SVMPs in its venom, and these structural variants (classified as P‐I, P‐II and P‐III SVMPs) can exhibit diverse functionalities (including being multi‐functional), ranging from haemorrhagic, fibrinogenolytic, Factor X or prothrombin activating activities to inhibiting platelet aggregation (Fox & Serrano, 2005; Casewell *et al*, 2015). Many SVMPs are haemorrhagic, with the P‐III class being broadly more haemorrhagic than the P‐IIs, and P‐IIs more so than the P‐Is (Fox & Serrano, 2005; Gutiérrez *et al*, 2010). Such SVMPs ultimately affect the integrity of endothelial cells in blood vessels, causing them to swell and burst and thereby promoting extravasation. However, this action is not due to direct cytotoxic effects, but instead by SVMPs binding and hydrolysing structural components of the basement membrane of capillary vessels, such as type IV collagen and perlecan, that link the membrane to the extracellular matrix (Gutiérrez *et al*, 2016). This cleavage weakens the scaffold structure and subsequently, haemodynamic forces (hydrostatic pressure and shear stress) induce a distention of the vessel wall, ultimately leading to rupture and haemorrhage (Gutiérrez *et al*, 2016).

Many SVMPs, particularly P‐Is, are fibrinogenolytic (Fox & Serrano, 2005). The majority of SVMPs actively cleave the α‐chain of fibrinogen into fibrinopeptides, and while some of these proteins also exhibit a lower degree of activity on the β‐chain, there are relatively few SVMPs that preferentially degrade the β‐chain (Markland, 1998). Ultimately, such degradation results in defibrination, which in turn contributes to coagulopathy and bleeding disturbances. Certain SVMPs have also been described as procoagulants by activating the clotting factors prothrombin or Factor X. Many of these toxins are derivations of P‐III SVMPs, and they are often found natively complexed to other venom toxins like C‐type lectins, although non‐complexed P‐I SVMPs with prothrombin activating capabilities have also been described (Fox & Serrano, 2005; Modesto *et al*, 2005; Gutiérrez *et al*, 2010). Factor X activators have been isolated from a number of snake venoms, including from both vipers and elapids, and the most well‐studied of these is the P‐III SVMP known as RVV‐X from the Russell's viper (*Daboia russelii*) (Siigur & Siigur, 2010). RVV‐X cleaves Factor X in the same manner as that which happens during the physiological activation of this clotting factor, resulting in the release of the serine protease known as activated Factor X (Factor Xa), which in turn acts on prothrombin in conjunction with activated Factor V (Factor Va) (Siigur & Siigur, 2010). RVV‐X requires the presence of Ca^2+^ for activation, as this induces a conformational change in Factor X that is a prerequisite for proteolysis by this SVMP (Siigur & Siigur, 2010). As described above, prothrombin is physiologically activated by the prothrombinase complex (Factor Xa and Factor Va), and this requires the presence of Ca^2+^ and phospholipases as cofactors. A number of SVMPs directly activate prothrombin to release thrombin, which in turn acts on fibrinogen to generate fibrin clotting. In doing so, these toxins (and also the Factor X activator RVV‐X) contribute strongly to VICC, by consuming the limited amounts of clotting factors physiologically available via their continual activation. Some SVMP‐based prothrombin activators are capable of functioning in the absence of cofactors, whereas others rely on the presence of calcium, and have thus been classified as either group A or group B prothrombin activators respectively (Rosing & Tans, 2010). Examples include the calcium‐independent activator ecarin, and the calcium‐dependent SVMP carinactivase‐1, both of which are found in the venom of the saw‐scaled viper *Echis carinatus* (Rosing & Tans, 2010).

Disintegrins are small (40–100 amino acid) cysteine‐rich polypeptides that are derived from the proteolytic cleavage of P‐II SVMPs or from genes solely encoding disintegrins (so called true or short‐coding disintegrins) (Calvete *et al*, 2005). Disintegrins are structurally diverse and best known for their action as integrin receptor antagonists, with different toxins capable of selectively blocking different cell‐surface integrins relevant to a variety of pathological conditions (e.g. α_2_β_3_ integrins for acute coronary ischaemia and thrombosis; α_v_β_3_ for tumour metastasis, osteoporosis and rheumatoid arthritis; α_4_β_1_, α_7_β_1_ and α_9_β_1_ for inflammation and autoimmune diseases, etc.) (Calvete *et al*, 2010). The most relevant to this review are those disintegrins that bind to α_2B_β_3_ integrins (the glycoprotein IIb/IIIa ‘platelet fibrinogen’ receptor), thereby preventing fibrinogen binding to platelets and inhibiting platelet aggregation (Calvete *et al*, 2005). These ‘RGD’ motif‐containing peptides have only been described from viperid snakes and it is not uncommon for such snakes to contain multiple different disintegrins in their venom (Calvete *et al*, 2010). In addition, some proteolytically processed P‐III SVMPs, consisting of disintegrin‐like domains coupled to cysteine‐rich domains, also appear to inhibit platelets via interactions with α_2_β_1_ integrins; resulting in the inhibition of collagen‐stimulated platelet aggregation (Shimokawa *et al*, 1997; Calvete *et al*, 2005). However, it remains unclear whether the disintegrin‐like domains of these toxins are responsible for this activity. In summary, SVMPs and related toxins exhibit various functions that impair haemostasis, including inducing haemorrhage, depleting various clotting factors and inhibiting platelet function. Their diversity and abundance in many snake venoms make them one of the most important toxin families relevant to venom‐induced haemotoxicity.

Snake venom serine proteases (SVSPs) are often referred to as thrombin‐like enzymes (TLEs) due to many exhibiting fibrinogenolytic functional activities analogous to thrombin. The majority of these serine protease enzymes have been isolated from viperid snakes, and many are known to effectively degrade fibrinogen into fibrinopeptides via proteolytic cleavage (Phillips *et al*, 2010). However, in contrast to thrombin, which readily cleaves both the α and β chains of fibrinogen, TLEs are typically more selective and usually only cleave one of these chains. Similarly to the SVMPs, most TLEs characterised to date act on the α chain, although there are examples of SVSPs that only cleave the β chain and a few that act on both α and β chains (Pirkle, 1998; Phillips *et al*, 2010). This cleavage of fibrinogen results in the polymerisation of fibrin monomers, but because TLEs do not stimulate factor XIII to cross‐link these polymers, as thrombin would, this results in unstable clots that are readily dissolved by plasmin (Phillips *et al*, 2010). Ultimately, the continual generation and destruction of fibrin thrombi results in a consumptive coagulopathy that depletes fibrinogen physiologically.

Thrombin‐like enzymes also exhibit other functionalities relevant to haemostasis by mimicking thrombin, which is itself a multi‐functional enzyme. For example, certain TLEs from the venom of the copperhead (*Agkistrodon contortrix contortrix*) and the horned desert viper (*Cerastes cerastes*) activate Factor XIII and either Factor V or Factor X, respectively (Marrakchi *et al*, 1995; Amiconi *et al*, 2000). Others induce the release and aggregation of platelets, potentially via their interaction with the protease‐activated receptor‐1 (PAR‐1) or the membrane glycoprotein receptor GpIb, both of which are found on the surface of platelets (Phillips *et al*, 2010). Certain TLE SVSPs have been described that exhibit kallikrein‐like functionalities by inducing the release of kinins, such as Lys‐bradykinin, by acting directly on plasma kininogens (Oyama & Takahashi, 2003). Finally, some TLEs are also capable of activating plasminogen by cleaving their peptide bonds, resulting in the release of plasmin and the degradation of fibrin, and therefore seem likely to contribute to consumption coaguloapathies via different mechanisms (Zhang *et al*, 1995).

There are two other types of serine protease toxins found in snake venom, and these are structurally and genetically distinct from the TLEs described above. Both of these serine proteases are only found in the venom of certain Australasian elapid snakes and they mimic activated versions of the clotting factors Factor X and Factor V, having evolved via the duplication of genes encoding these physiological blood clotting components (Reza *et al*, 2007; Reza & Kini, 2010). Consequently, these toxins activate prothrombin and are classified as group C and group D prothrombin activators (Rosing & Tans, 2010). Group C prothrombin activators, such as pseutarin C from the venom of the Eastern brown snake (*Pseudonaja textilis*) and oscutarin from the coastal taipan (*Oxyuranus scutellatus*), are large multi‐subunit proteases consisting of both Factor Xa‐like and Factor Va‐like subunits and therefore effectively mimic the prothrombinase complex (Rosing & Tans, 2010). These toxins are abundant in brown snake and taipan venom and they rely on Ca^2+^ and phospholipids to effectively activate prothrombin, resulting in the production of thrombin and the consumption of clotting factors, causing VICC (Reza *et al*, 2007; Isbister *et al*, 2010; Rosing & Tans, 2010; Maduwage & Isbister, 2014). Many more Australasian snakes have the less potent group D prothrombin activators in their venom. Similar to those described above, these toxins require Ca^2+^ and phospholipids as cofactors, but, unlike the group C activators, they also require the presence of Factor Va, as these toxins do not consist of multiple subunits and therefore do not mimic the prothrombinase complex, instead mimicking only activated Factor X (Reza & Kini, 2010; Rosing & Tans, 2010). As with group C activators, these toxins effectively cleave prothrombin into thrombin and initiate continual activation of the blood clotting cascade (Reza & Kini, 2010).

In addition to the SVMPs and SVSPs, there are many other snake venom toxin types that play a role in perturbing haemostasis. For example, certain kunitz‐type serine protease inhibitors found in venom (e.g. Textilinin‐1 and ‐2, also from the Eastern brown snake *P. textilis*) potently inhibit plasmin (and also thrombin), and thereby act as antifibrinolytic agents (Flight *et al*, 2005, 2009). Bradykinin‐potentiating peptides (BPPs), so called because they potentiate the effects of bradykinin, have been isolated from numerous pit viper species. These peptides have been demonstrated to inhibit angiotensin‐converting enzyme (ACE), thereby preventing the conversion of the hormone angiotensin I into the vasoconstrictor angiotensin II, resulting in a reduction in systemic blood pressure (Xu *et al*, 2015). Consequently, BPPs can contribute to hypotension observed following envenoming, and their effect is probably exacerbated by haemorrhagic SVMPs and SVSPs that exhibit kallikrein‐like functions (Fig 4A). Furthermore, certain snake venom PLA_2_s have also been demonstrated to induce hypotensive effects *in vivo*, and some of these functionally diverse proteins are also known to exert anticoagulant effects by inhibiting platelet aggregation (Andrião‐Escarso *et al*, 2002; Teixeira *et al*, 2011). Another toxin family known to affect platelet function are the C‐type lectin‐like proteins (CTLs). In fact, various CTLs have been described that exhibit a number of functional activities relating to haemostasis, including binding to Factor IX and X to inhibit blood coagulation, inhibiting the binding of thrombin to fibrinogen, and inhibiting or activating platelet aggregation by interacting with von Willebrand factor or collagen receptors (Lu *et al*, 2007; Arlinghaus *et al*, 2015).

## Pharmaceutical utility of snake venom haemotoxins

Venom components are gaining renewed interest as potential sources of new pharmacological compounds relevant for human therapeutics and diagnostics. Such developments are not limited to snake venoms, with toxins isolated from the toxic secretions of cone snails, sea anemones, spiders and scorpions exhibiting bioactivities relevant for the management of chronic pain, stroke and various autoimmune diseases (King, 2011). Nonetheless, snake venoms represent the most comprehensively studied of all venomous lineages and, consequently, have the most numerous lead compounds associated with them. Because of the specificity of many snake venom toxins, and their activity in targeting specific components related to cardiovascular processes, almost all of the snake venom derived pharmaceuticals available today have therapeutic or diagnostic indications relevant to haemostasis.

The first blockbuster drug developed from a venom toxin was Captopril (Capoten). Captropril was developed following the identification of an ACE inhibitor in the venom of the jararaca pit viper (*Bothrops jararaca*). This BPP inhibits the conversion of angiotensin I into angiotensin II, thereby reducing systemic blood pressure by preventing the production of a crucial vasoconstrictor (Ferreira *et al*, 1970). Consequently, captopril was developed in the 1970's as a synthetic analogue of this toxin and indicated for use as a hypotensive drug. It achieved US Food and Drug Administration approval in the early 1980's, subsequently becoming Squibb's first billion dollar drug, and the success of this product resulted in the development of many analogues (e.g. enalapril, lisinopril, perindopril and ramipril) being introduced into the market (Smith & Vane, 2003; McCleary *et al*, 2015).

Other snake venom toxin types have been successfully translated into human therapeutics. Both Tirofiban (Aggrastat) and Eptifibatide (Integrillin) are derived from disintegrin molecules found in the venoms of the saw‐scaled viper (*Echis carinatus*) and the dusky pygmy rattlesnake (*Sistrurus miliarius barbouri*) respectively. Both these disintegrins exhibit potent inhibition of the integrin receptor α_2_β_3_, thereby preventing the aggregation of platelets. Both toxins were developed synthetically; as a non‐peptidic molecule in the case of Tirofiban and as a cyclic heptapeptide analogue for Eptifibatide (McCleary *et al*, 2015). Consequently, these anticoagulant therapeutics function by effectively inhibiting platelet aggregation and they are indicated for use in patients suffering from unstable angina and myocardial infarctions (Peerlinck *et al*, 1993; Scarborough, 1999).

Batroxobin is a drug developed from a serine protease toxin (SVSP) isolated from the venom of the Brazilian lancehead viper (*Bothrops moojeni*). As with many SVSPs, this enzymatic toxin exhibits potent specificity for fibrinogen, in this case effectively releasing fibrinopeptide A via cleavage of the α‐chain of fibrinogen (McCleary *et al*, 2015). Such thrombin‐like SVSP enzymes also benefit from not being inhibited by classical serine protease inhibitors, whether endogenous (e.g. antithrombin III) or exogenous (e.g. hirudin) (Hutton & Warrell, 1993; McCleary *et al*, 2015). Although the fibrinogenolytic activity of batroxobin initially promotes the formation of clots, it is an anticoagulant drug of relevance for treating thrombotic disorders, as the degradation of fibrinogen leads to defibrination and, secondarily and indirectly, this in turn induces the release of tissue plasminogen activator, which converts plasminogen into plasmin and promotes the degradation of clots (McCleary *et al*, 2015). Specific indications for batroxobin include the treatment of ischaemic stroke, angina, myocardial and cerebral infraction and wound management after surgical interventions (Xu *et al*, 2007; Phillips *et al*, 2010).

Similarly, Ancrod (Arvin/Arwin/Viprinex), isolated from the venom of the Malayan pit viper (*Calloselasma rhodostoma*), is another well‐known SVSP that readily degrades fibrinogen, resulting in defibrination. Ancrod was also developed for use as an anticoagulant and indicated for the treatment of ischaemic stroke, myocardial infarction and deep‐vein thrombosis (Marsh & Williams, 2005), and showed potential benefit to patients suffering from heparin‐associated thrombocytopenia and thrombosis syndrome (Illig & Ouriel, 1996). However, over the course of many years equivocal results from clinical trials have since resulted in Ancrod being suspended from use (McCleary *et al*, 2015). Similarly, another defibrinating toxin, this time a synthetic form of an SVMP found in the venom of the copperhead (*Agkistrodon contortrix*), failed to meet desired endpoints in phase III clinical trials, despite positive results in phase I and II trials (Moll *et al*, 2006; Han *et al*, 2010). Consequently, the manufacturer withdrew this therapeutic, known as Alfimeprase, from further development.

Despite these setbacks, pharmaceutical manufacturers continue to pursue the development of venom components as future therapeutic agents for treating haemostatic disturbances. For example, other fibrinogenolytic SVSPs are currently in development, including ‘hemocogulase agkistrodon’ isolated from the Chinese moccasin (*Deinagkistrodon acutus*) and ‘crotalase’ from the Eastern diamondback rattlesnake (*Crotalus adamanteus*) (McCleary *et al*, 2015). Both of these serine protease toxins are in early development as anticoagulant therapeutics, with predicted indications including decreasing clotting times during surgical procedures (McCleary *et al*, 2015). Various venom toxins isolated from the venom of the Eastern brown snake (*P. textilis*) are also currently in development as haemostatic agents. The kunitz‐type serine protease inhibitor Textilinin‐1 inhibits plasmin, and has little‐to‐no effect on other serine proteases. This toxin therefore exhibits desirable characteristics as an anti‐fibrinolytic drug and, under the moniker Q8008, is being developed for use for reducing blood loss associated with complex surgeries (Earl *et al*, 2010). In addition, the activated Factor X‐ and Factor V‐like toxins found in *P. textilis* venom are also being translated into novel therapeutics to control bleeding. The Factor Xa‐like protein is being developed under the name of Haempatch to control bleeding at sites of trauma or surgery, whereas the Factor Va‐like protein, CoVase, is being assessed for its utility for combating non‐compressible haemorrhage (Earl *et al*, 2010; McCleary *et al*, 2015).

Haemotoxic components isolated from snake venoms are also commonly used for diagnostic purposes, particularly relating to blood clotting tests. For example, the SVMP toxin ecarin, a calcium independent prothrombin activator from the saw‐scaled viper (*E. carinatus*), has been used for over two decades as a standard in the ecarin clotting time test. This assay is used for the quantification of direct thrombin inhibitors, and has proven particularly useful as a means to monitor levels of drugs, such as hirudin, during anticoagulant therapy (Nowak, 2004). Ecarin is also used in a diagnostic test for lupus anticoagulants, alongside textarin from the Eastern brown snake (*P. textilis*) (Stocker *et al*, 1994). Using the variable specificity of these two procoagulant venom toxins, of which ecarin acts independently of cofactors whereas textarin requires phospholipids, calcium and factor V, to compare patient clotting times circumvents issues with lupus antibodies interfering with phospholipids, which other coagulation tests typically rely upon (Triplett *et al*, 1993). One such assay, the dilute Russell's viper venom time test (dRVVT test), also harnesses the procoagulant effect of snake venom as a clotting standard. In this case, coagulation is primarily the result of the SVMP Factor X activator RVV‐X and the SVSP Factor V activator RVV‐V and, consequently, Russell's viper venom has been used to assay blood clotting factors V and X, as well as lupus anticoagulants (Marsh & Williams, 2005). Finally, a protein C activating SVSP, protac, isolated from venom of the copperhead (*Agkistrodon contortrix*) (Stocker *et al*, 1987) is used in protein C and protein S diagnostic assays to test for activated protein C resistance, whereas the SVSP reptilase, from the lancehead viper (*Bothrops atrox*), is used to screen for heparin contamination in plasma, as its fibrinogenolytic activity is unaffected by heparin (Funk *et al*, 1971).

## Summary

Snake venom haemotoxins exhibit diverse functionalities that can result in haemorrhagic, coagulopathic and/or hypotensive pathology in snakebite victims. The functional diversity of these toxins and their relative abundance in many venoms, particularly those of vipers, mean that such haemotoxins are likely to act in a synergistic manner to perturb haemostasis. While providing an effective means for the snakes to catch their prey, for example by causing ischaemic or haemorrhagic events or shock due to systemic hypotension, the consequences for human snakebite victims can also be lethal. Consequently, furthering our understanding of the bioactivity of venom haemotoxins and their variation from one snake species to the next is essential for the design of next‐generation antivenom therapies. In addition, venom haemotoxins remain highly relevant for use as investigational ligands for understanding vertebrate physiology due to their high levels of selectivity and potency, and also for the development of new therapeutic and diagnostic pharmaceuticals relevant for human medicine.

## References

[bjh14591-bib-0001] Abubakar, I.S. , Abubakar, S.B. , Habib, A.G. , Nasidi, A. , Durfa, N. , Yusuf, P.O. , Larnyang, S. , Garnvwa, J. , Sokomba, E. , Salako, L. , Theakston, R.D.G. , Juszczak, E. , Alder, N. & Warrell, D.A. (2010) Randomised controlled double‐blind non‐inferiority trial of two antivenoms for saw‐scaled or carpet viper (*Echis ocellatus*) envenoming in Nigeria. PLoS Neglected Tropical Diseases, 4, e767. 20668549 10.1371/journal.pntd.0000767 PMC2910709

[bjh14591-bib-0002] Alirol, E. , Sharma, S.K. , Bawaskar, H.S. , Kuch, U. & Chappuis, F. (2010) Snake bite in South Asia: a review. PLoS Neglected Tropical Diseases, 4, e603. 20126271 10.1371/journal.pntd.0000603 PMC2811174

[bjh14591-bib-0003] Alirol, E. , Lechevalier, P. , Zamatto, F. , Chappuis, F. , Alcoba, G. & Potet, J. (2015) Antivenoms for snakebite envenoming: what is in the research pipeline? PLOS Neglected Tropical Diseases, 9, e0003896. 26355744 10.1371/journal.pntd.0003896 PMC4565558

[bjh14591-bib-0004] Amiconi, G. , Amoresano, A. , Boumis, G. , Brancaccio, A. , De Cristofaro, R. , De Pascalis, A. , Di Girolamo, S. , Maras, B. & Scaloni, A. (2000) A novel venombin B from *Agkistrodon contortrix contortrix*: evidence for recognition properties in the surface around the primary specificity pocket different from thrombin. Biochemistry, 39, 10294–10308. 10956019 10.1021/bi000145i

[bjh14591-bib-0005] Andrião‐Escarso, S.H. , Soares, A.M. , Fontes, M.R. , Fuly, A.L. , Corrêa, F.M. , Rosa, J.C. , Greene, L.J. & Giglio, J.R. (2002) Structural and functional characterization of an acidic platelet aggregation inhibitor and hypotensive phospholipase A2 from *Bothrops jararacussu* snake venom. Biochemical Pharmacology, 64, 723–732. 12167491 10.1016/s0006-2952(02)01210-8

[bjh14591-bib-0006] Antonypillai, C.N. , Wass, J.A.H. , Warrell, D.A. & Rajaratnam, H.N. (2011) Hypopituitarism following envenoming by Russell's vipers (*Daboia siamensis and D. russelii*) resembling Sheehan's syndrome: first case report from Sri Lanka, a review of the literature and recommendations for endocrine management. QJM, 104, 97–108. 21115460 10.1093/qjmed/hcq214

[bjh14591-bib-0007] Arlinghaus, F. , Fry, B. , Sunagar, K. , Jackson, T. , Eble, J. , Reeks, T. & Clemetson, K. (2015) Lectin proteins In: Venomous Reptiles & Their Toxins: Evolution, Pathophysiology & Biodiscovery (ed. by Fry B.), pp. 299–3311. Oxford University Press, New York.

[bjh14591-bib-0008] Arnold, C. (2016) Vipers, mambas and taipans: the escalating health crisis over snakebites. Nature, 537, 26–28. 27582205 10.1038/537026a

[bjh14591-bib-0009] Berling, I. , Brown, S.G.A. , Miteff, F. , Levi, C. & Isbister, G.K. (2015) Intracranial haemorrhages associated with venom induced consumption coagulopathy in Australian snakebites (ASP‐21). Toxicon, 102, 8–13. 26003794 10.1016/j.toxicon.2015.05.012

[bjh14591-bib-0010] Calvete, J.J. , Marcinkiewicz, C. , Monleón, D. , Esteve, V. , Celda, B. , Juárez, P. & Sanz, L. (2005) Snake venom disintegrins: evolution of structure and function. Toxicon, 45, 1063–1074. 15922775 10.1016/j.toxicon.2005.02.024

[bjh14591-bib-0011] Calvete, J. , Juarez, P. & Sanz, L. (2010) Snake venomics and disintegrins: potrait and evolution of a family of snake venom integrin antagonists In: Handbook of Venom and Toxins of Reptiles (ed. by Mackessy S.P.), pp. 337–357. CRC Press, Boca Raton.

[bjh14591-bib-0012] Camargo, A.C.M. , Ianzer, D. , Guerreiro, J.R. & Serrano, S.M.T. (2012) Bradykinin‐potentiating peptides: beyond captopril. Toxicon, 59, 516–523. 21835190 10.1016/j.toxicon.2011.07.013

[bjh14591-bib-0013] Casewell, N.R. , Cook, D.A.N. , Wagstaff, S.C. , Nasidi, A. , Durfa, N. , Wüster, W. & Harrison, R.A. (2010) Pre‐clinical assays predict pan‐African *Echis* viper efficacy for a species‐specific antivenom. PLoS Neglected Tropical Diseases, 4, e851. 21049058 10.1371/journal.pntd.0000851 PMC2964286

[bjh14591-bib-0014] Casewell, N.R. , Wagstaff, S.C. , Harrison, R.A. , Renjifo, C. , Wuster, W. & Wüster, W. (2011) Domain loss facilitates accelerated evolution and neofunctionalization of duplicate snake venom metalloproteinase toxin genes. Molecular Biology and Evolution, 28, 2637–2649. 21478373 10.1093/molbev/msr091

[bjh14591-bib-0015] Casewell, N.R. , Wüster, W. , Vonk, F.J. , Harrison, R.A. & Fry, B.G. (2013) Complex cocktails: the evolutionary novelty of venoms. Trends in Ecology & Evolution, 28, 219–229. 23219381 10.1016/j.tree.2012.10.020

[bjh14591-bib-0016] Casewell, N.R. , Wagstaff, S.C. , Wüster, W. , Cook, D.A.N. , Bolton, F.M.S. , King, S.I. , Pla, D. , Sanz, L. , Calvete, J.J. & Harrison, R.A. (2014) Medically important differences in snake venom composition are dictated by distinct postgenomic mechanisms. Proceedings of the National Academy of Sciences of the United States of America, 111, 9205–9210. 24927555 10.1073/pnas.1405484111 PMC4078820

[bjh14591-bib-0017] Casewell, N. , Sunagar, K. , Takacs, Z. , Calvete, J. , Jackson, T. & Fry, B. (2015) Snake venom metalloprotease enzymes In: Venomous Reptiles & Their Toxins: Evolution, Pathophysiology & Biodiscovery (ed. by Fry B.), pp. 347–363. Oxford University Press, New York.

[bjh14591-bib-0018] Chakrabarty, D. & Chanda, C. (2015) Snake venom disintegrins In: Snake Venoms, (ed. by Gopalkrishnakone P.), pp. 1–11. Living Reference Work, Springer, Dordrecht.

[bjh14591-bib-0019] Chippaux, J.P. (1998) Snakebites: appraisal of the global situation. Bulletin of the World Health Organisation, 76, 515–524. PMC2305789 9868843

[bjh14591-bib-0020] Chippaux, J.P. (2010) Snakebite in Africa: current situation and urgent needs In: Handbook of Venom and Toxins of Reptiles (ed. by Mackessy S.P.), pp. 453–474. CRC Press, Boca Raton.

[bjh14591-bib-0021] Chippaux, J.P. , Williams, V. & White, J. (1991) Snake venom variability: methods of study, results and interpretation. Toxicon, 29, 1279–1303. 1814005 10.1016/0041-0101(91)90116-9

[bjh14591-bib-0022] Cousin, X. , Bon, S. , Massoulié, J. & Bon, C. (1998) Identification of a novel type of alternatively spliced exon from the acetylcholinesterase gene of *Bungarus fasciatus*. Molecular forms of acetylcholinesterase in the snake liver and muscle. The Journal of Biological Chemistry, 273, 9812–9820. 9545320 10.1074/jbc.273.16.9812

[bjh14591-bib-0023] Deshpande, R.P. , Motghare, V.M. , Padwal, S.L. , Pore, R.R. , Bhamare, C.G. , Deshmukh, V.S. & Pise, H.N. (2013) Adverse drug reaction profile of anti‐snake venom in a rural tertiary care teaching hospital. Journal of Young Pharmacists, 5, 41–45. 24396245 10.1016/j.jyp.2013.02.003 PMC3828666

[bjh14591-bib-0024] Durban, J. , Pérez, A. , Sanz, L. , Gómez, A. , Bonilla, F. , Rodríguez, S. , Chacón, D. , Sasa, M. , Angulo, Y. , Gutiérrez, J.M. & Calvete, J.J. (2013) Integrated ‘omics’ profiling indicates that miRNAs are modulators of the ontogenetic venom composition shift in the Central American rattlesnake, Crotalus simus simus. BMC Genomics, 14, 234. 23575160 10.1186/1471-2164-14-234 PMC3660174

[bjh14591-bib-0025] Earl, S.T.H. , Masci, P.P. , De Jersey, J. , Lavin, M.F. & Dixon, J. (2010) Drug development from Australian elapid snake venoms and the Venomics pipeline of candidates for haemostasis: Textilinin‐1 (Q8008), Haempatch^TM^ (Q8009) and CoVase^™^ (V0801). Toxicon, 1, 1–8. 10.1016/j.toxicon.2010.12.010 21184772

[bjh14591-bib-0026] Escalante, T. , Rucavado, A. , Pinto, A.F.M. , Terra, R.M.S. , Gutiérrez, J.M. & Fox, J.W. (2009) Wound exudate as a proteomic window to reveal different mechanisms of tissue damage by snake venom toxins. Journal of Proteome Research, 8, 5120–5131. 19764775 10.1021/pr900489m

[bjh14591-bib-0027] Ferreira, S.H. , Bartelt, D.C. & Greene, L.J. (1970) Isolation of bradykinin‐potentiating peptides from *Bothrops jararaca* venom. Biochemistry, 9, 2583–2593. 4317874 10.1021/bi00815a005

[bjh14591-bib-0028] Flight, S. , Johnson, L. , Trabi, M. , Gaffney, P. , Lavin, M. , de Jersey, J. & Masci, P. (2005) Comparison of textilinin‐1 with aprotinin as serine protease inhibitors and as antifibrinolytic agents. Pathophysiology of Haemostasis and Thrombosis, 34, 188–193. 16707925 10.1159/000092421

[bjh14591-bib-0029] Flight, S.M. , Johnson, L.A. , Du, Q.S. , Warner, R.L. , Trabi, M. , Gaffney, P.J. , Lavin, M.F. , de Jersey, J. & Masci, P.P. (2009) Textilinin‐1, an alternative anti‐bleeding agent to aprotinin: importance of plasmin inhibition in controlling blood loss. British Journal of Haematology, 145, 207–211. 19236611 10.1111/j.1365-2141.2009.07605.x

[bjh14591-bib-0030] Fox, J.W. & Serrano, S.M.T. (2005) Structural considerations of the snake venom metalloproteinases, key members of the M12 reprolysin family of metalloproteinases. Toxicon, 45, 969–985. 15922769 10.1016/j.toxicon.2005.02.012

[bjh14591-bib-0031] Fry, B.G. , Wüster, W. , Kini, R.M. , Brusic, V. , Khan, A. , Venkataraman, D. & Rooney, A.P. (2003) Molecular evolution and phylogeny of elapid snake venom three‐finger toxins. Journal of Molecular Evolution, 57, 110–129. 12962311 10.1007/s00239-003-2461-2

[bjh14591-bib-0032] Fry, B.G. , Scheib, H. , van der Weerd, L. , Young, B. , McNaughtan, J. , Ramjan, S.F.R. , Vidal, N. , Poelmann, R.E. & Norman, J.A. (2008) Evolution of an arsenal: structural and functional diversification of the venom system in the advanced snakes (Caenophidia). Molecular & Cellular Proteomics, 7, 215–246. 17855442 10.1074/mcp.M700094-MCP200

[bjh14591-bib-0033] Funk, C. , Gmür, J. , Herold, R. & Straub, P.W. (1971) Reptilase‐R ‐ a new reagent in blood coagulation. British Journal of Haematology, 21, 43–52. 5105276 10.1111/j.1365-2141.1971.tb03415.x

[bjh14591-bib-0034] Greene, H.W. (1997) Snakes : The Evolution of Mystery in Nature. University of California Press, Berkeley.

[bjh14591-bib-0035] Gutiérrez, J.M. , Rucavado, A. & Escalante, T. (2010) Snake venom metalloproteinases: biological roles and participation in the pathophysiology of envenoming In: Handbook of Venom and Toxins of Reptiles (ed. by Mackessy S.P.), pp. 115–137. CRC Press, Boca Raton.

[bjh14591-bib-0036] Gutiérrez, J.M. , Escalante, T. , Rucavado, A. & Herrera, C. (2016) Hemorrhage caused by snake venom metalloproteinases: a journey of discovery and understanding. Toxins, 8, 93. 27023608 10.3390/toxins8040093 PMC4848620

[bjh14591-bib-0037] Habib, A.G. , Kuznik, A. , Hamza, M. , Abdullahi, M.I. , Chedi, B.A. , Chippaux, J.‐P. & Warrell, D.A. (2015) Snakebite is under appreciated: appraisal of burden from West Africa. PLOS Neglected Tropical Diseases, 9, e0004088. 26398046 10.1371/journal.pntd.0004088 PMC4580425

[bjh14591-bib-0038] Han, S.M. , Weaver, F.A. , Comerota, A.J. , Perler, B.A. & Joing, M. (2010) Efficacy and safety of alfimeprase in patients with acute peripheral arterial occlusion (PAO). Journal of Vascular Surgery, 51, 600–609. 20110153 10.1016/j.jvs.2009.08.053

[bjh14591-bib-0039] Harrison, R. & Gutiérrez, J. (2016) Priority actions and progress to substantially and sustainably reduce the mortality, morbidity and socioeconomic burden of tropical snakebite. Toxins, 8, 351. 10.3390/toxins8120351 PMC5198546 27886134

[bjh14591-bib-0040] Harrison, R.A. , Hargreaves, A. , Wagstaff, S.C. , Faragher, B. & Lalloo, D.G. (2009) Snake envenoming: a disease of poverty. PLoS Neglected Tropical Diseases, 3, e569. 20027216 10.1371/journal.pntd.0000569 PMC2791200

[bjh14591-bib-0041] Harrison, R.A. , Cook, D.A. , Renjifo, C. , Casewell, N.R. , Currier, R.B. & Wagstaff, S.C. (2011) Research strategies to improve snakebite treatment: challenges and progress. Journal of Proteomics, 74, 1768–1780. 21723969 10.1016/j.jprot.2011.06.019

[bjh14591-bib-0042] Hutton, R.A. & Warrell, D.A. (1993) Action of snake venom components on the haemostatic system. Blood Reviews, 7, 176–189. 8241832 10.1016/0268-960x(93)90004-n

[bjh14591-bib-0043] Illig, K.A. & Ouriel, K. (1996) Ancrod: understanding the agent. Seminars in Vascular Surgery, 9, 303–314. 8958607

[bjh14591-bib-0044] Isbister, G. (2010) Snakebite doesn't cause disseminated intravascular coagulation: coagulopathy and thrombotic microangiopathy in snake envenoming. Seminars in Thrombosis and Hemostasis, 36, 444–451. 20614396 10.1055/s-0030-1254053

[bjh14591-bib-0045] Isbister, G.K. , Scorgie, F.E. , O'Leary, M.A. , Seldon, M. , Brown, S.G.A. & Linz, L.F. (2010) Factor deficiencies in venom‐induced consumption coagulopathy resulting from Australian elapid envenomation: Australian Snakebite Project (ASP‐10). Journal of Thrombosis and Haemostasis, 8, 2504–2513. 20831619 10.1111/j.1538-7836.2010.04050.x

[bjh14591-bib-0046] Isbister, G.K. , Maduwage, K. , Scorgie, F.E. , Shahmy, S. , Mohamed, F. , Abeysinghe, C. , Karunathilake, H. , O'Leary, M.A. , Gnanathasan, C.A. & Lincz, L.F. (2015) Venom concentrations and clotting factor levels in a prospective cohort of Russell's viper bites with coagulopathy. PLoS Neglected Tropical Diseases, 9, e0003968. 26296235 10.1371/journal.pntd.0003968 PMC4546603

[bjh14591-bib-0047] Kasturiratne, A. , Wickremasinghe, A.R. , de Silva, N. , Gunawardena, N.K. , Pathmeswaran, A. , Premaratna, R. , Savioli, L. , Lalloo, D.G. & de Silva, H.J. (2008) The global burden of snakebite: a literature analysis and modelling based on regional estimates of envenoming and deaths. PLoS Medicine, 5, e218. 18986210 10.1371/journal.pmed.0050218 PMC2577696

[bjh14591-bib-0048] Katkar, G.D. , Sundaram, M.S. , NaveenKumar, S.K. , Swethakumar, B. , Sharma, R.D. , Paul, M. , Vishalakshi, G.J. , Devaraja, S. , Girish, K.S. & Kemparaju, K. (2016) NETosis and lack of DNase activity are key factors in *Echis carinatus* venom‐induced tissue destruction. Nature Communications, 7, 11361. 10.1038/ncomms11361 PMC4838891 27093631

[bjh14591-bib-0049] Kerkkamp, H.M.I. , Casewell, N.R. & Vonk, F.J. (2015) Evolution of the Snake Venom Delivery System In: Evolution of Venomous Animals and Their Toxins, (ed. by Gopalkrishnakone P. & Malhotra A.), pp. 1–11. Living Reference Work, Springer, Dordrecht.

[bjh14591-bib-0050] King, G.F. (2011) Venoms as a platform for human drugs: translating toxins into therapeutics. Expert Opinion on Biological Therapy, 11, 1469–1484. 21939428 10.1517/14712598.2011.621940

[bjh14591-bib-0051] Kini, R. & Koh, C. (2016) Metalloproteases affecting blood coagulation, fibrinolysis and platelet aggregation from snake venoms: definition and nomenclature of interaction sites. Toxins, 8, 284. 10.3390/toxins8100284 PMC5086644 27690102

[bjh14591-bib-0052] Lu, Q. , Clemetson, J.M. & Clemetson, K.J. (2007) Snake venom C‐type lectins interacting with platelet receptors. Toxin Reviews, 26, 77–93. 10.1016/j.toxicon.2005.02.022 15876445

[bjh14591-bib-0053] Lynch, V.J. (2007) Inventing an arsenal: adaptive evolution and neofunctionalization of snake venom phospholipase A2 genes. BMC Evolutionary Biology, 7, 2. 17233905 10.1186/1471-2148-7-2 PMC1783844

[bjh14591-bib-0054] Maduwage, K. & Isbister, G.K. (2014) Current treatment for venom‐induced consumption coagulopathy resulting from snakebite. PLoS Neglected Tropical Diseases, 8, e3220. 25340841 10.1371/journal.pntd.0003220 PMC4207661

[bjh14591-bib-0055] Markland, F.S. (1998) Snake venom fibrinogenolytic and fibrinolytic enzymes: an updated inventory. Registry of Exogenous Hemostatic Factors of the Scientific and Standardization Committee of the International Society on Thrombosis and Haemostasis. Thrombosis and Haemostasis, 79, 668–674. 9531060

[bjh14591-bib-0056] Marrakchi, N. , Zingali, R.B. , Karoui, H. , Bon, C. & el Ayeb, M. (1995) Cerastocytin, a new thrombin‐like platelet activator from the venom of the Tunisian viper *Cerastes cerastes* . Biochimica et Biophysica Acta, 1244, 147–156. 7766651 10.1016/0304-4165(94)00216-k

[bjh14591-bib-0057] Marsh, N. & Williams, V. (2005) Practical applications of snake venom toxins in haemostasis. Toxicon, 45, 1171–1181. 15922782 10.1016/j.toxicon.2005.02.016

[bjh14591-bib-0058] Massey, D.J. , Calvete, J.J. , Sánchez, E.E. , Sanz, L. , Richards, K. , Curtis, R. & Boesen, K. (2012) Venom variability and envenoming severity outcomes of the *Crotalus scutulatus scutulatus* (Mojave rattlesnake) from Southern Arizona. Journal of Proteomics, 75, 2576–2587. 22446891 10.1016/j.jprot.2012.02.035

[bjh14591-bib-0059] McCleary, R.J.R. , Kang, T.S. & Kini, R.M. (2015) Reptile venoms as a platform for drugdevelopment In: Venoms to Drugs: Venoms as a Source for the Development of Human Therapeutics (ed. by King G.F.), pp. 129–162. Royal Society of Chemistry, Cambridge.

[bjh14591-bib-0060] Modesto, J.C. , Junqueira‐de‐Azevedo, I.L.M. , Neves‐Ferreira, A.G.C. , Fritzen, M. , Oliva, M.L.V. , Ho, P.L. , Perales, J. & Chudzinski‐Tavassi, A.M. (2005) Insularinase A, a prothrombin activator from *Bothrops insularis* venom, is a metalloprotease derived from a gene encoding protease and disintegrin domains. Biological Chemistry, 386, 589–600. 16006246 10.1515/BC.2005.069

[bjh14591-bib-0061] Moll, S. , Kenyon, P. , Bertoli, L. , De Maio, J. , Homesley, H. & Deitcher, S.R. (2006) Phase II trial of alfimeprase, a novel‐acting fibrin degradation agent, for occluded central venous access devices. Journal of Clinical Oncology, 24, 3056–3060. 16809729 10.1200/JCO.2006.05.8438

[bjh14591-bib-0062] Mosquera, A. , Idrovo, L.A. , Tafur, A. & Del Brutto, O.H. (2003) Stroke following *Bothrops spp*. snakebite. Neurology, 60, 1577–1580. 12771244 10.1212/01.wnl.0000061614.52580.a1

[bjh14591-bib-0063] Moura‐da‐Silva, A.M. , Furlan, M.S. , Caporrino, M.C. , Grego, K.F. , Portes‐Junior, J.A. , Clissa, P.B. , Valente, R.H. & Magalhães, G.S. (2011) Diversity of metalloproteinases in *Bothrops neuwiedi* snake venom transcripts: evidences for recombination between different classes of SVMPs. BMC Genetics, 12, 94. 22044657 10.1186/1471-2156-12-94 PMC3217872

[bjh14591-bib-0064] Nowak, G. (2004) The ecarin clotting time, a universal method to quantify direct thrombin inhibitors. Pathophysiology of Haemostasis and Thrombosis, 33, 173–183. 10.1159/000081505 15583446

[bjh14591-bib-0065] Oyama, E. & Takahashi, H. (2003) Purification and characterization of a thrombin like enzyme, elegaxobin II, with lys‐bradykinin releasing activity from the venom of *Trimeresurus elegans* (Sakishima‐Habu). Toxicon, 41, 559–568. 12676434 10.1016/s0041-0101(02)00363-x

[bjh14591-bib-0066] Peerlinck, K. , De Lepeleire, I. , Goldberg, M. , Farrell, D. , Barrett, J. , Hand, E. , Panebianco, D. , Deckmyn, H. , Vermylen, J. & Arnout, J. (1993) MK‐383 (L‐700,462), a selective nonpeptide platelet glycoprotein IIb/IIIa antagonist, is active in man. Circulation, 88, 1512–1517. 8403299 10.1161/01.cir.88.4.1512

[bjh14591-bib-0067] Phillips, D. , Swenson, S. & Markland, F. Jr (2010) Thrombin‐like snake venom serine proteases In: Handbook of Venom and Toxins of Reptiles (ed. by Mackessy S.P.), pp. 139–154. CRC Press, Boca Raton.

[bjh14591-bib-0068] Pirkle, H. (1998) Thrombin‐like enzymes from snake venoms: an updated inventory. Scientific and Standardization Committee's Registry of Exogenous Hemostatic Factors. Thrombosis and Haemostasis, 79, 675–683. 9531061

[bjh14591-bib-0069] Pla, D. , Paiva, O.K. , Sanz, L. , Beutler, M. , Wright, C.E. , Calvete, J.J. , Williams, D.J. & Gutiérrez, J.M. (2014) Preclinical efficacy of Australian antivenoms against the venom of the small‐eyed snake, *Micropechis ikaheka*, from Papua New Guinea: an antivenomics and neutralization study. Journal of Proteomics, 110, 198–208. 24980637 10.1016/j.jprot.2014.06.016

[bjh14591-bib-0070] Reza, M.A. & Kini, R. (2010) Origin and evolution of snake vneom prothrombin activators In: Toxins and Haemostasis: From Bench to Bedside (eds. by Kini R., Clemetson K., Markland F., McLane M. & Morita T.), pp. 501–518. Springer, Dordrecht.

[bjh14591-bib-0071] Reza, M.A. , Swarup, S. & Kini, R.M. (2007) Structure of two genes encoding parallel prothrombin activators in *Tropidechis carinatus* snake: gene duplication and recruitment of factor X gene to the venom gland. Journal of Thrombosis and Haemostasis, 5, 117–126. 17239167 10.1111/j.1538-7836.2006.02266.x

[bjh14591-bib-0072] Rivel, M. , Solano, D. , Herrera, M. , Vargas, M. , Villalta, M. , Segura, Á. , Arias, A.S. , León, G. & Gutiérrez, J.M. (2016) Pathogenesis of dermonecrosis induced by venom of the spitting cobra, *Naja nigricollis*: an experimental study in mice. Toxicon, 119, 171–179. 27288896 10.1016/j.toxicon.2016.06.006

[bjh14591-bib-0073] Rosing, J. & Tans, G. (2010) Snake venom prothrombin activators ‐ the history In: Toxins and Haemostasis: From Bench to Bedside (eds. by Kini R., Clemetson K., Markland F., McLane M. & Morita T.), pp. 485–499. Springer, Dordrecht.

[bjh14591-bib-0074] Rucavado, A. , Soto, M. , Escalante, T. , Loría, G.D. , Arni, R. & Gutiérrez, J.M. (2005) Thrombocytopenia and platelet hypoaggregation induced by *Bothrops asper* snake venom: toxins involved and their contribution to metalloproteinase‐induced pulmonary hemorrhage. Thrombosis and Haemostasis, 94, 123–131. 16113795 10.1160/TH05-02-0112

[bjh14591-bib-0075] Scarborough, R.M. (1999) Development of eptifibatide. American Heart Journal, 138, 1093–1104. 10577440 10.1016/s0002-8703(99)70075-x

[bjh14591-bib-0076] Segura, Á. , Villalta, M. , Herrera, M. , León, G. , Harrison, R. , Durfa, N. , Nasidi, A. , Calvete, J.J. , Theakston, R.D.G. , Warrell, D.A. & Gutiérrez, J.M. (2010) Preclinical assessment of the efficacy of a new antivenom (EchiTAb‐Plus‐ICP^®^) for the treatment of viper envenoming in sub‐Saharan Africa. Toxicon, 55, 369–374. 19699756 10.1016/j.toxicon.2009.08.010

[bjh14591-bib-0077] Shimokawa, K. , Shannon, J.D. , Jia, L.G. & Fox, J.W. (1997) Sequence and biological activity of catrocollastatin‐C: a disintegrin‐like/cysteine‐rich two‐domain protein from *Crotalus atrox* venom. Archives of Biochemistry and Biophysics, 343, 35–43. 9210644 10.1006/abbi.1997.0133

[bjh14591-bib-0078] Siigur, J. & Siigur, E. (2010) Activation of Factor X by snake venom proteases In: Toxins and Haemostasis: From Bench to Bedside (eds. by Kini R., Clemetson K., Markland F., McLane M. & Morita T.), pp. 447–464. Springer, Dordrecht.

[bjh14591-bib-0079] Silva, A. , Kuruppu, S. , Othman, I. , Goode, R.J.A. , Hodgson, W.C. & Isbister, G.K. (2017) Neurotoxicity in Sri Lankan Russell's Viper (*Daboia russelii*) Envenoming is Primarily due to U1‐viperitoxin‐Dr1a, a Pre‐Synaptic Neurotoxin. Neurotoxicity Research. 31, 11–19. 27401825 10.1007/s12640-016-9650-4

[bjh14591-bib-0080] Smith, C.G. & Vane, J.R. (2003) The discovery of captopril. FASEB Journal, 17, 788–789. 12724335 10.1096/fj.03-0093life

[bjh14591-bib-0081] Stocker, K. , Fischer, H. , Meier, J. , Brogli, M. & Svendsen, L. (1987) Characterization of the protein C activator Protac from the venom of the southern copperhead (*Agkistrodon contortrix*) snake. Toxicon, 25, 239–252. 3590209 10.1016/0041-0101(87)90253-4

[bjh14591-bib-0082] Stocker, K. , Hauer, H. , Müller, C. & Triplett, D.A. (1994) Isolation and characterization of TextarinR, a prothrombin activator from eastern brown snake (*Pseudonaja textilis*) venom. Toxicon, 32, 1227–1236. 7846693 10.1016/0041-0101(94)90352-2

[bjh14591-bib-0083] Tan, C. , Tan, N. , Tan, K. & Kwong, K. (2015) Antivenom cross‐neutralization of the venoms of *Hydrophis schistosus* and *Hydrophis curtus*, two common sea snakes in Malaysian waters. Toxins, 7, 572–581. 25690691 10.3390/toxins7020572 PMC4344642

[bjh14591-bib-0084] Tan, C.H. , Liew, J.L. , Tan, K.Y. & Tan, N.H. (2016a) Genus *Calliophis* of Asiatic coral snakes: a deficiency of venom cross‐reactivity and neutralization against seven regional elapid antivenoms. Toxicon, 121, 130–133. 27616455 10.1016/j.toxicon.2016.09.003

[bjh14591-bib-0085] Tan, C.H. , Liew, J.L. , Tan, K.Y. & Tan, N.H. (2016b) Assessing SABU (Serum Anti Bisa Ular), the sole Indonesian antivenom: a proteomic analysis and neutralization efficacy study. Scientific Reports, 6, 37299. 27869134 10.1038/srep37299 PMC5116744

[bjh14591-bib-0086] Teixeira, S.S. , Silveira, L.B. , da Silva, F.M.N. , Marchi‐Salvador, D.P. , Silva, F.P. , Izidoro, L.F.M. , Fuly, A.L. , Juliano, M.A. , dos Santos, C.R. , Murakami, M.T. , Sampaio, S.V. , da Silva, S.L. & Soares, A.M. (2011) Molecular characterization of an acidic phospholipase A2 from *Bothrops pirajai* snake venom: synthetic C‐terminal peptide identifies its antiplatelet region. Archives of Toxicology, 85, 1219–1233. 21331602 10.1007/s00204-011-0665-6

[bjh14591-bib-0087] Triplett, D.A. , Stocker, K.F. , Unger, G.A. & Barna, L.K. (1993) The Textarin/Ecarin ratio: a confirmatory test for lupus anticoagulants. Thrombosis and Haemostasis, 70, 925–931. 8165613

[bjh14591-bib-0088] Vidal, N. , Rage, J.‐C. , Couloux, A. & Hedges, S.B. (2009) Snakes (Serpentes) In: The Timetree of Life (eds. by Hedges S.B. & Kumar S.), pp. 390–397. Oxford University Press, New York.

[bjh14591-bib-0089] Visser, L.E. , Kyei‐Faried, S. , Belcher, D.W. , Geelhoed, D.W. , van Leeuwen, J.S. & van Roosmalen, J. (2008) Failure of a new antivenom to treat *Echis ocellatus* snake bite in rural Ghana: the importance of quality surveillance. Transactions of the Royal Society of Tropical Medicine and Hygiene, 102, 445–450. 18190937 10.1016/j.trstmh.2007.11.006

[bjh14591-bib-0090] Vonk, F.J. , Admiraal, J.F. , Jackson, K. , Reshef, R. , de Bakker, M.A.G. , Vanderschoot, K. , van den Berge, I. , van Atten, M. , Burgerhout, E. , Beck, A. , Mirtschin, P.J. , Kochva, E. , Witte, F. , Fry, B.G. , Woods, A.E. & Richardson, M.K. (2008) Evolutionary origin and development of snake fangs. Nature, 454, 630–633. 18668106 10.1038/nature07178

[bjh14591-bib-0091] Vonk, F.J. , Casewell, N.R. , Henkel, C.V. , Heimberg, A.M. , Jansen, H.J. , McCleary, R.J.R. , Kerkkamp, H.M.E. , Vos, R.A. , Guerreiro, I. , Calvete, J.J. , Wüster, W. , Woods, A.E. , Logan, J.M. , Harrison, R.A. , Castoe, T.A. , de Koning, A.P.J. , Pollock, D.D. , Yandell, M. , Calderon, D. , Renjifo, C. , Currier, R.B. , Salgado, D. , Pla, D. , Sanz, L. , Hyder, A.S. , Ribeiro, J.M. , Arntzen, J.W. , van den Thillart, G.E. , Boetzer, M. , Pirovano, W. , Dirks, R.P. , Spaink, H.P. , Duboule, D. , McGlinn, E. , Kini, R.M. & Richardson, M.K. (2013) The king cobra genome reveals dynamic gene evolution and adaptation in the snake venom system. Proceedings of the National Academy of Sciences of the United States of America, 110, 20651–20656. 24297900 10.1073/pnas.1314702110 PMC3870661

[bjh14591-bib-0092] Warrell, D. (1995a) Clinical Toxicology of Snakebite in Africa and the Middle East/Arabian Peninsula In: Handbook of Clinical Toxicology of Animal Venoms and Poisons (eds. by White J. & Meier J.), pp. 455–492. CRC Press, Boca Raton.

[bjh14591-bib-0093] Warrell, D. (1995b) Clinical toxicology of snakebite in Asia In: Handbook of Clinical Toxicology of Animal Venoms and Poisons (eds. by White J. & Meier J.), pp. 534–594. CRC Press, Boca Raton.

[bjh14591-bib-0094] Williams, D.J. (2015) Snake bite: a global failure to act costs thousands of lives each year. BMJ, 351, h5378. 26508421 10.1136/bmj.h5378

[bjh14591-bib-0095] Williams, D.J. , Gutiérrez, J.‐M. , Calvete, J.J. , Wüster, W. , Ratanabanangkoon, K. , Paiva, O. , Brown, N.I. , Casewell, N.R. , Harrison, R.A. , Rowley, P.D. , O'Shea, M. , Jensen, S.D. , Winkel, K.D. & Warrell, D.A. (2011) Ending the drought: new strategies for improving the flow of affordable, effective antivenoms in Asia and Africa. Journal of Proteomics, 74, 1735–1767. 21640209 10.1016/j.jprot.2011.05.027

[bjh14591-bib-0096] World Health Organization (2010a) Guidelines for the prevention and clinical management of snakebite in Africa. Available at: http://apps.who.int/iris/bitstream/10665/204458/1/9789290231684.pdf?ua=1 (accessed 16th December 2016).

[bjh14591-bib-0097] World Health Organization (2010b) Guidelines for the management of snake‐bite: South East Asia. Available at: http://apps.who.int/iris/bitstream/10665/204464/1/B4508.pdf?ua=1 (Accessed 16th December 2016).

[bjh14591-bib-0098] Xu, G. , Liu, X. , Zhu, W. , Yin, Q. , Zhang, R. & Fan, X. (2007) Feasibility of treating hyperfibrinogenemia with intermittently administered batroxobin in patients with ischemic stroke/transient ischemic attack for secondary prevention. Blood Coagulation & Fibrinolysis, 18, 193–197. 17287638 10.1097/MBC.0b013e328040c0f2

[bjh14591-bib-0099] Xu, X. , Li, B. , Zhu, S. & Rong, R. (2015) Hypotensive peptides from snake venoms: structure, function and mechanism. Current Topics in Medicinal Chemistry, 15, 658–669. 25686732 10.2174/1568026615666150217113835

[bjh14591-bib-0100] Zhang, Y. , Wisner, A. , Xiong, Y. & Bon, C. (1995) A novel plasminogen activator from snake venom. Purification, characterization, and molecular cloning. The Journal of Biological Chemistry, 270, 10246–10255. 7730329 10.1074/jbc.270.17.10246

